# Metallothionein-I + II Reduces Oxidative Damage and Apoptosis after Traumatic Spinal Cord Injury in Rats

**DOI:** 10.1155/2018/3265918

**Published:** 2018-11-05

**Authors:** Camilo Rios, Iván Santander, Marisela Méndez-Armenta, Concepción Nava-Ruiz, Sandra Orozco-Suárez, Marcela Islas, Verónica Barón-Flores, Araceli Diaz-Ruiz

**Affiliations:** ^1^Departamento de Sistemas Biológicos, Universidad Autónoma Metropolitana Unidad Xochimilco Ciudad de México, Mexico; ^2^Departamento de Neuroquímica, Instituto Nacional de Neurología y Neurocirugía Manuel Velasco Suárez, Ciudad de México, Mexico; ^3^Laboratorio de Patología Experimental, Instituto Nacional de Neurología y Neurocirugía Manuel Velasco Suárez, Ciudad de México, Mexico; ^4^Unidad de Investigación Médica en Enfermedades Neurológicas, Hospital de Especialidades, Centro Médico Nacional Siglo XXI, Ciudad de México, Mexico

## Abstract

After spinal cord injury (SCI), some self-destructive mechanisms start leading to irreversible neurological deficits. It is known that oxidative stress and apoptosis play a major role in increasing damage after SCI. Metallothioneins I and II (MT) are endogenous peptides with known antioxidant, neuroprotective capacities. Taking advantage of those capacities, we administered exogenous MT to rats after SCI in order to evaluate the protective effects of MT on the production of reactive oxygen species (ROS) and lipid peroxidation (LP), as markers of oxidative stress. The activities of caspases-9 and -3 and the number of annexin V and TUNEL-positive cells in the spinal cord tissue were also measured as markers of apoptosis. Rats were subjected to either sham surgery or SCI and received vehicle or two doses of MT (10 *μ*g per rat) at 2 and 8 h after surgical procedure. The results showed a significant increase in levels of MT protein by effect of SCI and SCI plus treatment at 12 h, while at 24 h an increase of MT was observed only in the injury plus treatment group (*p* < 0.05). ROS production was decreased by effect of MT in lesioned tissue; likewise, we observed diminished LP levels by MT effect both in the sham group and in the group with SCI. Also, the results showed an increase in the activity of caspase-9 due to SCI, without changes by effect of MT, as compared to the sham group. Caspase-3 activity was increased by SCI, and again, MT treatment reduced this effect only at 24 h after injury. Finally, the results of the number of cells positive to annexin V and TUNEL showed a reduction due to MT treatment both at 24 and 72 h after the injury. With the findings of this work, we conclude that exogenously administered MT has antioxidant and antiapoptotic effects after SCI.

## 1. Introduction

After spinal cord injury (SCI), a series of self-destructive mechanisms initiate to produce irreversible damage to the surrounding tissue, with consequent motor and sensitive deficits [1]. Among those damaging mechanisms, the ischemia after trauma with subsequent energy failure and ATP deficit [2] produces depolarization of the plasma membrane leading, in turn, to increased intracellular calcium by opening calcium voltage-activated channels. Excessive calcium entry to cells produces reactive oxygen and nitrogen species (ROS and RNS, respectively). The ROS include superoxide anion (O_2_•^−^), hydrogen peroxide (H_2_O_2_), and hydroxyl radical (^•^OH). O_2_•^−^ is produced through several pathways during normal metabolism, and superoxide dismutase (SOD) enzymes convert O_2_^•−^ into H_2_O_2_. H_2_O_2_ is reduced to H_2_O by catalase, glutathione peroxidase (GPx), and thioredoxin [3]. Likewise, nitric oxide (•NO) synthesized by the activation of constitutive and inducible nitric oxide synthases after SCI [4, 5] can react with O_2_^•−^ to form the highly reactive oxidizing agent, peroxynitrite (ONOO–). The increased production of ROS and RNS after SCI cause oxidative damage to proteins, DNA, and cellular lipids, polyunsaturated fatty acids in cell membranes, triggering free radical chain reactions to cause lipid peroxidation (LP) [6]. Furthermore, damage to proteins and DNA activates the mechanisms of cell death by apoptosis [7]. Apoptosis occurs through intrinsic and extrinsic apoptotic pathways. The intrinsic one starts when mitochondria are exposed to a pathological overload of calcium; opening of the mitochondrial permeability transition pore (mPTP) is triggered, activating the initiator caspase-9 and subsequently activating the effector caspase-3 [8]. Based on this information, it has been proposed that the prevention of apoptosis after damage could be a key target to preventing spinal cord tissue damage and to promoting improved motor function after SCI. On the other hand, we have reported that metallothioneins (MTs) could play an important role in regulating oxidative damage [9]. They are known to be nonenzymatic intracellular proteins of low molecular weight, consisting of 61–62 amino acid residues, and high content of cysteines (25–30%). They form disulfide bridges and have high affinity for metals, binding 5–7 zinc atoms, 12 copper atoms, or 7 atoms of cadmium per mole of protein [9]. Three protein isoforms (MT-I, MT-II, and MT-III) have been identified in the central nervous system [9]; from these, MT-I and MT-II are located in the central nervous system and peripheral tissues; MT-III isoform is expressed specifically in the brain and spinal cord [10] neurons. A common feature observed in numerous studies is the remarkable ability of MT-I and II to reduce cell death and oxidative brain damage [11]. MTs are known to have high antioxidant capacity, even greater than that of glutathione, and this antioxidant mechanism has been proposed as responsible for its neuroprotective effect [12]. MT easily reacts with •OH, O2•^−^, and •NO radicals, and thiol groups can bind ONOO– and peroxynitrous acid (ONOOH), making this protein a highly efficient antioxidant defense [12–14]. Likewise, the neuroprotective effect of MT has been reported to reduce apoptosis in transgenic mice overexpressing MT. They showed reduced cell death and oxidative tissue damage after traumatic brain injury [15]. Finally, it has been reported that MT-III attenuated the apoptosis of neurons in the CA1 region of the hippocampus in a model of Alzheimer's disease in mice [16]. In the present study, we evaluated the antioxidant and antiapoptotic effects of MT in a model of SCI contusion in rats.

## 2. Materials and Methods

### 2.1. Animals

We used female Wistar rats weighing 200 to 250 g of body weight; they were maintained under standard laboratory conditions and had free access to food and water, following the guidelines established internationally and nationally by the Mexican Official Standard NOM-062-ZOO-1999 (which observes technical specifications for the production, care, and use of laboratory animals) and the guidelines for Care and Use of Laboratory Animals of the National Institutes of Health (USA). API MT-I + II with a zinc content about 6%, from rabbit liver, purity ≥95%, was purchased from Creative BioMart.

### 2.2. Surgical Procedure

All animals were subjected to either a sham procedure (laminectomy only) or spinal cord injury (SCI) by contusion according to the report of Basso et al. [17], under pentobarbital (50 mg/kg i.p.) anesthesia. An incision was made by extending from the mid to low thoracic regions, followed by a laminectomy, including the caudal portion of T9 and all of T10, to expose the spinal cord. The NYU weight-drop device was used in order to produce the SCI by contusion. Then, the SCI was produced by dropping the 10 g rod from a distance of 25 mm. The surgical site was sutured in layers. Rats were allowed to recover from anesthetic and surgical procedures in an intensive care unit for small animals (Schroer Manufacturing Co., Kansas City, MO, USA), and the animals received food and water *ad libitum*. Their intestine and bladder were manually expressed twice a day, and they were routinely inspected visually for skin irritation and decubitus ulcers.

### 2.3. Experimental Design and Treatment with MT-I + II

The rats were randomly assigned to either one of the following four groups: group 1, sham surgery plus vehicle i.p. (saline solution, 0.9% NaCl); group 2, sham surgery plus MT i.p. (metallothionein); group 3, animals with SCI plus vehicle; and group 4, rats with SCI plus treatment with MT. The MT was exogenously i.p. administered at doses of 10 *μ*g per rat, dissolved in saline solution, according to the previous reports by Penkowa and Hidalgo [18], starting two and eight hours after SCI, following the schedule reported previously (Diaz-Ruiz et al. [19]). According to that report, a dose of 10 *μ*g is enough to produce therapeutic benefits, while a higher dose showed no further neuroprotective effect.

### 2.4. Metallothionein Protein Assay

Seventy-three animals were sacrificed at two different times (12 and 24) after surgical procedure. The content of MT was estimated by the silver-saturation method as described by Scheuhammer and Cherian [20] and modified by Rojas and Ríos [21]. Briefly, fresh tissue samples of about 0.02 g were homogenized in 300 *μ*l of a 0.05 M phosphate buffer (pH = 7) and 0.375 M NaCl mixture (1.5 : 1 *v*/*v*). Then, 250 *μ*l of silver nitrate solution (20 ppm) and 400 *μ*l of glycine buffer (0.5 M, pH 8.5) were added. After standing at room temperature for 5 min, 100 *μ*l of rat hemolyzed erythrocytes was added and the mixture was boiled for 2 min, then centrifuged at 4000 x/g for 5 min. The latter step was repeated twice. MT content was estimated by measuring the silver content of the supernatant fractions (diluted 1 : 10 with 3% HNO_3_*v*/*v*) using an atomic absorption spectrophotometer (Perkin-Elmer 3110) equipped with an HGA-600 furnace and AS60 autosampler. The results were expressed as nmol of metallothionein per gram of wet tissue.

### 2.5. Reactive Oxygen Species Assay

To evaluate the amount of reactive oxygen species (ROS) in the spinal cord tissue, thirty-one animals were sacrificed (prior anesthesia) at 4 h after surgery procedure, as reported by Liu et al. [22], as the time for increased production of hydrogen peroxide and superoxide radical. ROS were assayed following the method previously reported by Pérez-Severiano et al. [23]. The samples of fresh tissue were taken at the level of the ninth thoracic vertebra (a single sample from each rat), 2 mm from the caudal area and 2 mm from the rostral area, covering only the epicenter of the lesion or laminectomy. Tissue was homogenized in 20 volumes of saline solution (0.9% NaCl), and aliquots of 100 *μ*l were incubated in the presence of 5 *μ*M 2′,7′-dichlorodihydrofluorescein diacetate (DCF) at 37°C for 60 min. Fluorescent signals were recorded at the end of the incubation time at an excitation wavelength of 488 nm and an emission wavelength of 525 nm in a Perkin-Elmer LS50B luminescence spectrometer. A standard curve was constructed using increasing concentrations of DCF incubated in parallel. Results were expressed as nmol of 2′,7′-dichlorodihydrofluorescein diacetate (DCF) per gram of wet tissue per one hour of incubation.

### 2.6. Lipid Peroxidation Assay

The LP was assessed as a marker of oxidative damage, using lipid-fluorescence product formation as a marker. Forty-two animals were killed by decapitation 24 h after either SCI or sham surgery, when the peak of LP levels is reached, according to Diaz-Ruiz et al. [6]. The samples of wet tissue were taken in the same way as described above. Lipid-soluble fluorescent products (LFP), an index of LP, were measured according to the technique described by Triggs and Willmore [24] and modified by Santamaría et al. [25]. Briefly, each sample of spinal cord tissue was homogenized in 3 ml of saline solution (0.9% NaCl). One-milliliter aliquots of the homogenate were added to 4 ml of a chloroform-methanol mixture (2 : 1, *v*/*v*). After stirring, the mixture was ice-cooled for 30 min to allow phase separation and the fluorescence of the chloroform layer was measured in a Perkin-Elmer LS50B luminescence spectrophotometer at 370 nm (excitation) and 430 nm (emission). The sensitivity of the spectrophotometer was adjusted to 150 fluorescence units with a quinine standard solution (0.1 g/ml). The values were expressed as fluorescent units per gram of wet tissue.

### 2.7. Activities of Caspases-9 and -3

To characterize the antiapoptotic effect of MT, the activities of caspases 9 and 3 were measured. Ninety-four rats were killed at 24 and 72 hours according to reports by Ríos et al. [7] as the times for maximal activity of the caspases after SCI. Animals were killed by decapitation, and one centimeter (5 mm from the caudal area and 5 mm from the rostral area) of spinal cord wet tissue was obtained from each rat. Caspase-9 activity was measured by using a caspase-9 fluorometric protease ELISA Kit that recognizes the sequence LEHD (Chemicon MCH6 kit) [7]. The assay detects the cleavage of LEHD-AFC (7-amino-4-trifluoromethyl coumarin), as a substrate. LEHD-AFC emits blue light that was detected at 400 nm; upon cleavage of the substrate by MCH-6 or related caspases, free AFC emits a yellow-green fluorescence at 505 nm, which was quantified by using a fluorescence plate reader (BioTek model FLX 800TB). Comparison of the fluorescence of AFC from an apoptotic sample with an uninduced control allowed us to determine the increase in caspase-9 activity.

Caspase-3 activity was measured by using a Calbiochem Caspase-3 Activity Assay Kit. The enzyme cleaves specifically after aspartate residues in a different peptide sequence (DEVD). The DEVD substrate is also labeled with the fluorescent molecule AFC, so the reaction can be monitored by a blue to green shift in fluorescence upon cleavage of the AFC fluorophore [26]. The results were expressed in arbitrary fluorescence units per milligram of protein (measured according to Lowry, 1951) of 5 to 8 animals per groups.

### 2.8. Annexin V and TUNEL Immunodetection

Thirty-five animals were killed at either 24 and 72 h after SCI to assess the antiapoptotic effect of MT by immunohistochemical technique. Approximately 1 cm (5 mm from the caudal area and 5 mm from the rostral area) of the spinal cord at the T9 level was removed immediately after cardiac perfusion with 0.1 M phosphate buffered saline (PBS) and 4% paraformaldehyde solution in PBS and post-fixed for one week at 4°C and processed for embedding in paraffin. Longitudinal sections were then cut (12 *μ*m thickness) with the aid of a microtome (Leica RM2125 RT, Germany) and mounted onto poly-l-lysine-coated slides. To have a comparable area for evaluation, the ependymus was taken as the point of reference, and the area with the most tissue destruction was considered to be the epicenter of the injury. Only this section was considered for the analysis. Then, the sections were washed in 0.12 M phosphate buffer saline (PBS, pH 7.2–7.6) and incubated for 30 min at room temperature with blocking solution, 1% normal horse serum (Vector Lab) in PBS 0.12 M. Later, the sections were incubated with anti-annexin V (Santa Cruz Labs, SC-8300) antibody diluted in PBS (1 : 200) and neurofilament (Santa Cruz Labs, SC-32273) for 24 h at 4°C. After that, the sections were washed with PBS 0.12 M for 15 min and incubated for 2 h at room temperature with the goat anti-mouse IgG Alexa 488 and Alexa 546 (Molecular Probes, Invitrogen Lab, A21121, A11030) diluted in PBS. In the next step, the sections were counterstained with Hoechst (Invitrogen) or propidium iodide (Sigma) for 1 min and a final washing was performed in PBS 0.12 M for 10 min, gently dried, and cover-slipped with Vectashield (Vector Lab) mounting medium. The samples were observed in a fluorescence microscope (Carl Zeiss), with refrigerated camera (Evolution) and an image analyzer Image-Pro Plus 7 (Media Cybernetics).

### 2.9. Analysis of the Number of Annexin V- and TUNEL-Positive Cells

Annexin V- and TUNEL-positive cells were counted in the cross-longitudinal sections of the spinal cord at the lesion site. All images were digitized using an Evolution MP freeze camera (Media Cybernetics, USA) connected to an Axio Lab microscope (Zeiss, Germany) and Image-Pro Plus 7 software to analyze the images and count the cells. The average cell density per unit volume was determined with the optical fractionator method [27]. This procedure allowed the determination of the fraction of tissue in which neurons were counted. Every third section was sampled (for a total of 10 sections). Then, the first sampling fraction was 1/3; this is called the section sampling fraction. A volume fraction of each tissue was taken, and the area sampling fraction (asf) = area (frame)/area (*x* · *y*) was the area of counting frame (220 × 180 *μ*m), relative to the area associated with each field in the computer monitor. The third sampling fraction reflected that cells were not counted in the entire thickness of the tissue at each sampling location. Instead, a three-dimensional probe of a known height was placed in the tissue. The thickness of the tissue (6 *μ*m) divided by the height of the dissector was the third sampling fraction. This is called the tissue sampling fraction or tsf. The estimate of the total cell number was therefore the sum of cells counted (Σ*Q*–), multiplied by the reciprocal of the three fractions of the brain region sampled as represented by

(1) $$\begin{array}{c} N\frac{1}{4}\sum Q-\left(\frac{1}{ssf}\right)\left(\frac{1}{asf}\right)\left(tsf\right), \end{array}$$

where *N* is the estimate of the total cell number and Σ*Q*– is the number of counted cells on all sections. In order to standardize the counting, the same volume fraction was used for each experimental group. This method was standardized and validated by West [27] and modified by Besio et al. [28].

### 2.10. Statistical Analysis

An exploratory analysis of the data was performed to determine normal distribution (Kolmogorov-Smirnov's test) and homogeneity of variances, applying Levene's test. The results of MT protein levels, amount of ROS, and activities of caspases-9 and -3 were analyzed applying one-way ANOVA, followed by Tukey's test for multiple comparisons. Data of annexin V (24 and 72 h) and TUNEL-positive cells (24 h) were analyzed using Student's *t*-test. The results of the LP- and TUNEL-positive cell (only 72 h) counting were analyzed using the Kruskal-Wallis test, followed by Mann-Whitney's *U* test for multiple comparisons, due to lack of normal distribution and homogeneity of variances of data. All analyses were performed with an SPSS 22.0 software. Differences were considered statistically significant when *p* < 0.05.

## 3. Results

### 3.1. Both the Lesion and Treatment with MT Increased the Concentration of Metallothionein Proteins after SCI

**Figure 1:**
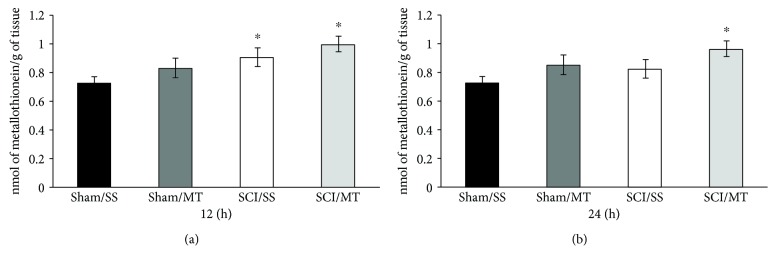
Spinal cord tissue metallothionein (MT) levels of rats submitted to either spinal cord injury or sham operation and evaluated at 12 (a) and 24 h (b) after surgery and treated with saline solution (SS) or MT (10 *μ*g/rat). Sham: rats without spinal cord injury; SCI: rats at two times after spinal cord injury. The results are expressed as mean ± SEM of 8 to 11 animals per group, ^∗^different from the sham/SS group (*p* < 0.05). One-way ANOVA followed by Tukey's test.

The content of metallothionein proteins present in the spinal cord tissue was assessed at 12 (Figure 1(a)) and 24 h (Figure 1(b)) following either laminectomy (sham) or SCI from animals receiving either vehicle (saline solution) or MT (10 *μ*g/rat) at 2 and 8 h after surgical procedure. The results are shown as mean values ± SEM and are expressed as nmol of metallothionein per gram of tissue. Figure 1(a) shows an average baseline value of 0.723 ± 0.042 (*n* = 8) of metallothionein in intact animals that received vehicle (sham/SS) while the mean values for sham injury and MT was 0.831 ± 0.066 (*n* = 9); no significant differences were observed (*p* = 0.23). Likewise, the mean value of rats with SCI and vehicle (SCI/SS) was 0.911 ± 0.063 (*n* = 10) and the value from rats with lesion plus MT (SCI/MT) was of 0.999 ± 0.055 (*n* = 8); both values were statistically significant as compared to the sham/SS group. Also, Figure 1(b) shows the mean values ± S.E.M. of MT content in animals without lesion with vehicle or MT (0.727 ± 0.049 (*n* = 11) and 0.852 ± 0.053 (*n* = 9), respectively) while average values ± S.E.M. of injury groups treated with either vehicle or MT were 0.826 ± 0.040 (*n* = 10) and 0.96 ± 0.064 (*n* = 8), respectively; only the sham/SS vs. SCI/MT group showed statistically significant differences (*p* = 0.004).

### 3.2. Exogenous Metallothionein Reduces the Production of Reactive Oxygen Species after SCI

**Figure 2:**
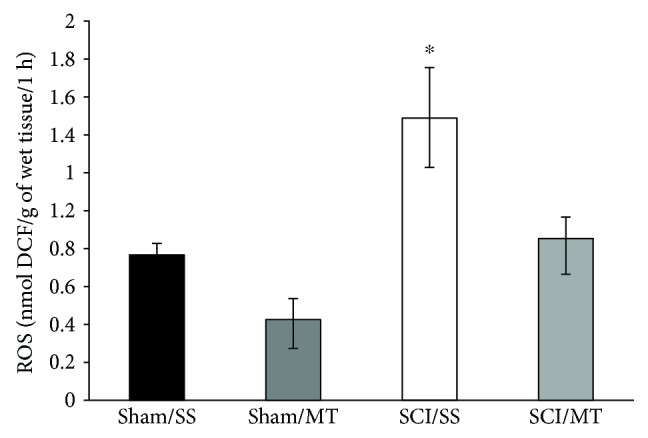
Formation of reactive oxygen species (ROS) spinal cord tissue, 4 h after surgery procedure. The results are expressed as mean ± SEM of nmol of 2′,7′-dichlorofluorescein diacetate (DCF) formed per grams of wet tissue per hour of 6 to 8 animals per group. Sham/SS: animals with laminectomy only plus saline solution and Sham/MT: rats with laminectomy with metallothionein (10 *μ*g per rat) at 2 and 8 h surgical procedure. SCI/SS: spinal cord injury and treatment with saline solution; SCI/MT: spinal cord contusion and treatment with metallothionein. One-way ANOVA following Tukey's test, ^∗^*p* < 0.05, different from all groups.

Shown in Figure 2 are the results of the amount of reactive oxygen species (ROS) present in the spinal cord tissue of animals either treated or not with MT. The values are expressed as mean ± SEM of *n* = 28 animals. The results are given as nmol of 2′,7′-dichlorofluorescein (DCF) formed per gram of wet tissue per hour. As observed, the values of ROS in group sham/SS was 0.76 ± 0.06 (*n* = 6), while the sham/MT group was 0.42 ± 0.11 (*n* = 8); likewise, the value in the SCI/SS group was 1.49 ± 0.27 (*n* = 6) and 0.86 ± 0.11 for the SCI/MT group (*n* = 8). The results show an increase in the amount of ROS due to the damage, which is reversed by MT treatment (*p* < 0.05). Finally, we did not find statistically significant differences due to the effect of MT on the noninjured tissue. One-way ANOVA followed Tukey's test.

### 3.3. Metallothionein Reduces Tissue Lipid Peroxidation in Both Healthy and Damaged Tissue

**Figure 3:**
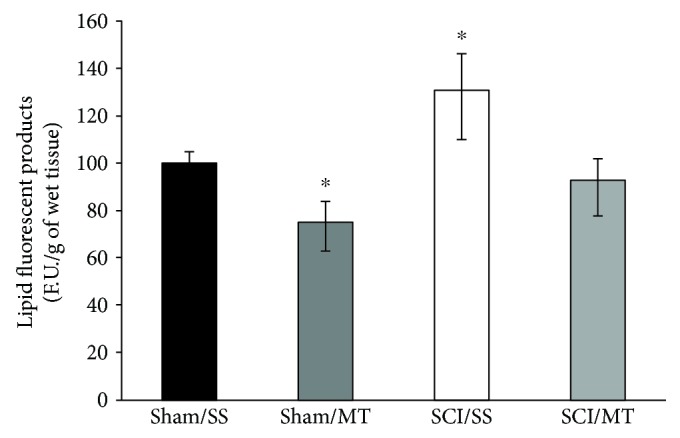
Effect of metallothionein (MT) upon lipid peroxidation (LP) levels evaluated 4 h after surgical procedure in rats. The results are expressed as means ± SEM of florescence units per gram of wet tissue of 9 to 14 animals per group. Sham/SS: animals with laminectomy only plus saline solution; Sham/MT: rats with laminectomy with MT (10 *μ*g per rat) at 2 and 8 h after surgery; SCI/SS: spinal cord injury and treatment with saline solution; SCI/MT: spinal cord contusion and treatment with metallothionein. Kruskal-Wallis following Mann-Whitney's *U* test, ^∗^*p* < 0.05, different of all groups; ^∗∗^*p* < 0.05 different vs. SCI/SS.

The results of LP levels after sham and SCI procedure are shown in Figure 3. The values are expressed as mean ± SEM of *n* = 42 animals and are expressed in fluorescence units per gram of wet tissue. The average values observed in group sham/SS was 99.58 ± 7.4 (*n* = 14), while in the sham group treated with MT, the mean value was reduced to 75.02 ± 5.47 (*n* = 9); this reduction was statistically significant (*p* < 0.05). Likewise, in the groups with SCI without treatment (SCI/SS), there was an increase in the LP of 132.50 ± 8.47 (*n* = 9) and the group treated with MT (SCI/MT) showed a statistically significant reduction to 94.96 ± 5.33 (*n* = 10). The results show that the treatment with MT has an antioxidant effect, since it was able to reduce the LP in both tissues submitted only to the surgical procedure and after damage. The Kruskal-Wallis test was used following Mann-Whitney's *U* test.

### 3.4. Exogenous Metallothionein Did Not Change Caspase-9 Activity after Spinal Cord Injury

**Figure 4:**
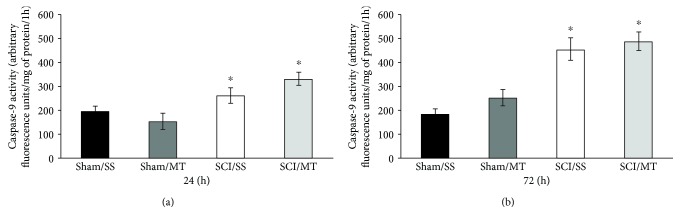
Caspase-9 activity measured in spinal cord tissue at 24 (a) and 72 h (b) after surgery procedure. Sham/SS: animals with laminectomy only plus saline solution; Sham/MT: rats with laminectomy with MT (10 *μ*g per rat) at 2 and 8 h after surgery; SCI/SS: spinal cord injury and treatment with saline solution; SCI/MT; spinal cord contusion and treatment with metallothionein at 2 and 8 h after damage. The results are expressed in arbitrary fluorescence units per mg of protein per 1 h and correspond to the average value ± SEM of 5 to 8 animals per group, ^∗^different from the Sham/SS group (*p* < 0.05). One-way ANOVA followed by Tukey's test.

To assess the intrinsic apoptotic pathway, we measured the activity of caspase-9 at 24 (Figure 4(a)) and 72 h (Figure 4(b)) following laminectomy (sham) or SCI.

The results are shown as mean values ± S.E.M. and are expressed as florescence units per mg of protein per 1-hour incubation. Figure 4(a) shows a mean of 190.67 ± 21.02 (*n* = 6) of caspase-9 activity in sham-injury animals receiving vehicle (sham/SS), while the mean value for sham-injury plus MT was 143.40 ± 11.00 (*n* = 5). No significant differences were observed. Likewise, rats with SCI and vehicle (SCI/SS) showed a mean of 250.94 ± 28.35 (*n* = 8) and the value from rats with lesion plus MT (SCI/MT) was 325.50 ± 32.72 (*n* = 5); both values were statistically significant as compared to the sham/SS group.

Figure 4(b) shows the mean values ± S.E.M. of caspase-9 activity 72 h after the surgical procedure. Animals without lesion, with vehicle or MT treatment, showed values of 178.80 ± 14.12 (*n* = 5) and 244.50 ± 26.67 (*n* = 5), respectively, while average values ± SEM of injury groups treated with vehicle or MT were of 451.42 ± 61.35 (*n* = 6) and 485.58 ± 54.09 (*n* = 6), respectively. No differences were found among groups by effect of MT treatment.

### 3.5. Treatment with Exogenous MT Diminished Caspase-3 Activity Only 24 h after Surgical Procedure

**Figure 5:**
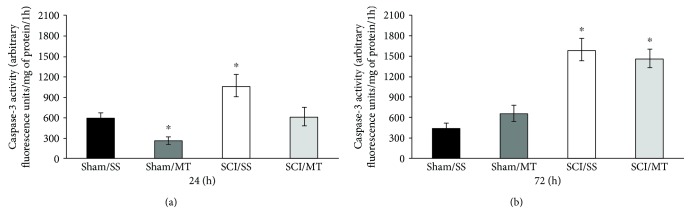
Caspase-3 activity measured in spinal cord tissue at 24 (a) and 72 h (b) after surgery procedure. Sham/SS: animals with laminectomy only plus saline solution; Sham/MT: rats with laminectomy with MT (10 *μ*g per rat) at 2 and 8 h after surgery; SCI/SS: spinal cord injury and treatment with saline solution; SCI/MT: spinal cord contusion and treatment with metallothionein at 2 and 8 h after damage. The results are expressed in arbitrary fluorescence units per mg of protein per 1 h and correspond to the average value ± SEM of 4 to 9 animals per group, ^∗^different from the Sham/SS group (*p* < 0.05). One-way ANOVA followed by Tukey's test.

The results of the activity of caspase-3 assessed at 24 and 72 h following laminectomy (sham) or spinal cord contusion injury model (SCI) are shown in Figures 5(a) and 5(b). The results are shown as mean values ± SEM and are expressed as florescence units per mg of protein per 1 hour.  Figure 5(a) shows an average value of 580.29 ± 56.03 (*n* = 7) of caspase-3 activity from animals without injury receiving vehicle (sham/SS), while the mean value for sham-injury plus MT was 270.71 ± 41.18 (*n* = 7) observing a statistically significant reduction of due to MT treatment (*p* < 0.05). Likewise, rats with SCI plus vehicle (SCI/SS) showed a mean of 1048.94 ± 101.22 (*n* = 8); this increase was different vs. the sham/SS group (*p* < 0.05). Likewise, the value from rats with SCI plus MT (SCI/MT) was 678.44 ± 64.64 (*n* = 9), a reduction vs. SCI/SS. Also, Figure 5(b) shows the mean values ± S.E.M. of caspase-3 activity, 72 h after surgical procedure. Animals without lesion plus vehicle or MT showed values of 418.38 ± 56.81 (*n* = 4) and 625.10 ± 60.03 (*n* = 5), respectively. Meanwhile, average values ± S.E.M. of injury groups treated with either vehicle or MT were 1573.75 ± 204.75 (*n* = 4) and 1473.25 ± 213.91 (*n* = 4), respectively, with a statistical reduction in caspase-3 activity (*p* < 0.05) as compared to the sham/SS group.

### 3.6. Metallothionein Reduces the Number of Annexin V-Positive Cells after Spinal Cord Injury

**Figure 6:**
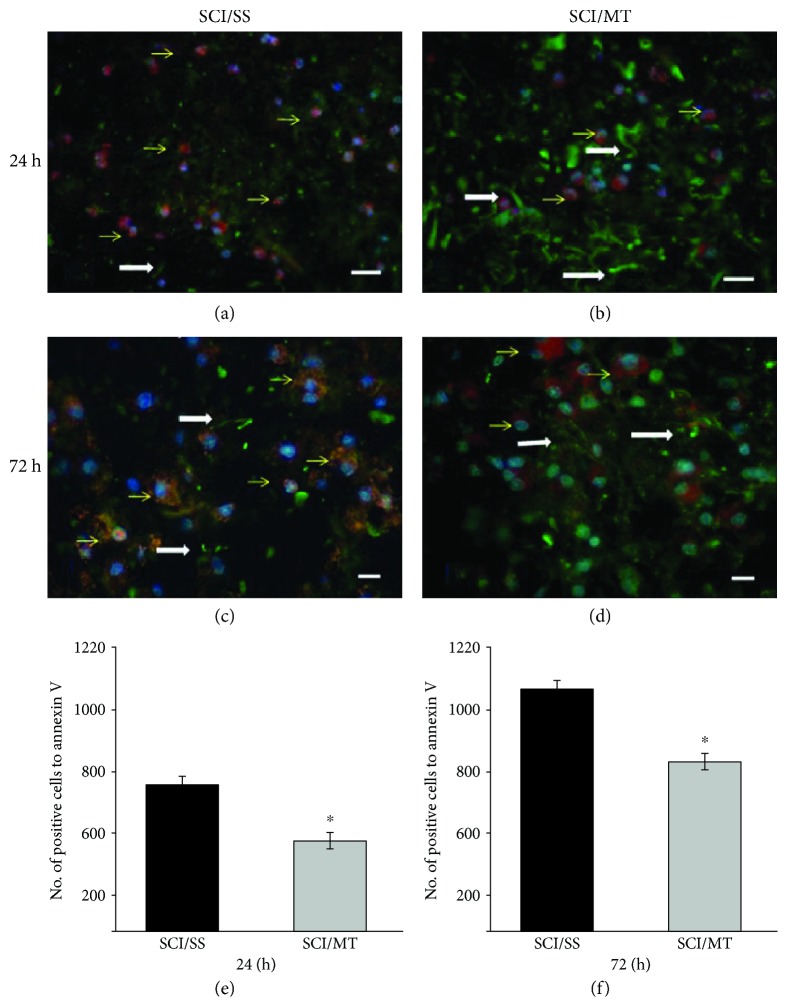
Representative microphotographs of annexin V-positive cells (red, Alexa 546) and neurofilaments (green, Alexa 488) in the injury area after spinal cord contusion and sacrificed at 24 or 72 hours after damage. (a, c) Animals with spinal cord injury (SCI) plus saline solution (SS); (b, d) rats with SCI and treated with two doses of metallothionein (MT) starting at 2 and 8 h after the lesion; all animals were sacrificed at 24 h after SCI. (a, b) Animals with similar conditions described above for SS and (c) and SCI plus MT but sacrificed 72 hours after injury. Yellow arrows showed annexin V-reactive cells, as well as some immunoreactive fibers to NF (white arrows); the MT reduced neuronal damage and preserved a greater number of fibers, Scale bar 20 *μ*m. (e, f): Number of annexin V-positive cells per mm^3^ measured at 24 or 72 h, respectively, after damage. The results are shown as average values ± SEM of 3–6 animals per group. Student's *T* test, ^∗^*p* < 0.05.

Figures 6(a)–6(d) show representative images of annexin V-positive cells analyzed at 24 and 72 h after injury of animals with SCI treated with vehicle or MT. Likewise, Figures 6(e) and 6(f) show the number of cells positive to Annexin V from rats with SCI and treated either with vehicle or with MT and evaluated at 24 or 72 h after damage. The results are expressed as the number of cells positive to Annexin V per mm^3^ and are expressed as the average value ± SEM of 3 to 6 animals per group. As can be observed, there is a decrease in the number of annexin V-positive cells in animals both killed at 24 h and at 72 and treated with MT (548 ± 16.68 and 809 ± 37.31, respectively) as compared to SCI plus vehicle evaluated at those times (747 ± 46.11 and 1069 ± 46.41); these reductions were 26.64 and 25.32%, respectively, and were statistically significant (*p* < 0.05).

### 3.7. Metallothionein Reduces the Number of TUNEL-Positive Cells after Spinal Cord Injury, at 72 h after SCI

**Figure 7:**
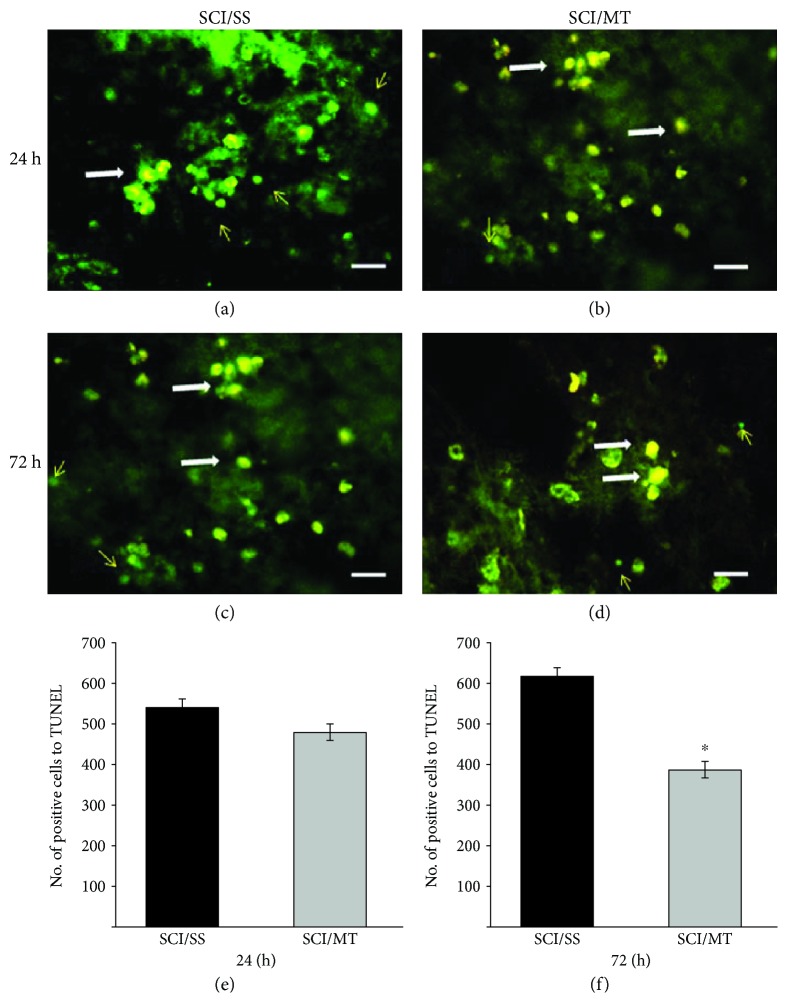
Representative microphotographs of TUNEL-positive cells in the injury area after spinal cord contusion and sacrificed at 24 or 72 hours after damage. (a, c) Animals with spinal cord injury (SCI) plus saline solution (SS); (b, d) rats with SCI and treated with two doses of metallothionein (MT) starting at 2 and 8 h after the lesion; all animals were sacrificed at 24 h after SCI. (a, b) Animals with similar condition described above for SS and panels: SCI plus MT but sacrificed 72 hours after injury. Arrows showed TUNEL-positive cells (bright green), and orange cells showed TUNEL and propidium iodide which means that they die by necrosis (white arrow). Scale bar 20 *μ*m. (e, f) Number of TUNEL-positive cells per mm^3^ measured at 24 or 72 h, respectively, after damage. The results are shown as average values ± SEM of 3–6 animals per group. Mann-Whitney's *U* test, ^∗^*p* < 0.05.

Figures 7(a)–7(d) show representative images of TUNEL-positive cells observed at 24 and 72 h after injury. Representative images from animals with SCI plus vehicle and rats lesioned plus MT are shown. Likewise, Figures 7(e) and 7(f) show the counting of TUNEL-positive cells evaluated 24 and 72 h after lesion, respectively. As observed, a nonsignificant decrease in the number of TUNEL-positive cells was found in the animals treated with SCI plus MT as compared SCI/SS, evaluated at 24 h after the damage. Meanwhile, animals killed 72 h after injury showed a mean of 612 ± 40.56 in SCI animals treated with vehicle, while TUNEL-positive cells were 389 ± 11.88 in animals treated with SCI plus MT. This difference reached significance (*p* < 0.05) and represents a 36.44% reduction.

## 4. Discussion

The results of the present work showed that MT increased in the injured tissue of rats treated or not with MT, only at short times (12 h) after injury. At 24 h, only animals treated with exogenously administered MT continue with increased levels of MT protein. This increase in MT proteins by exogenously administered MT seems to reduce the amount of ROS (only in damage tissue) while this antioxidant effect is also observed as a reduction of LP both in animals from the sham group and in the lesioned group (SCI/SS). As the effect of exogenously administered MT is observed both in the sham-injury and SCI animals, we can conclude that MT is exerting a direct antioxidant effect on tissue, even in the basal conditions of the cells, but only in the LP biomarker. The antioxidant effect of MT may be due to the large number of thiol groups present in the MT molecule. Cai and Cherian [12] showed that MT exerts a greater antioxidant effect than glutathione, accompanied by a cytoprotective effect. Likewise, it has been described that MT reacts easily with hydroxyl radical, superoxide radical, and nitric oxide, while thiol groups from MT clusters A and B can bind anion peroxynitrite and peroxynitric acid, making MT a highly efficient antioxidant defense molecule [13, 14]. In studies carried out in cultures of modified cells overexpressing MT-I and II, it is possible to observe a clear protection against oxidative stress conditions, in opposition to what happens if those proteins are not expressed [29, 30]. In addition, the antioxidant capacity of MT has been tested in models of damage to the central nervous system, where MT reduced lipoperoxidation and nitration of tyrosine generated by peroxynitrite formation [9] Recently, Duan et al. [31] showed that MT treatment protected against Cd-induced hepatotoxicity, as a result of the antioxidant effect of the protein.

On the other hand, this study demonstrated that MT has no effect on the activity of caspase-9 at all times evaluated, suggesting that the intrinsic pathway is not involved in the antiapoptotic effect of MT, observed both as a reduction of caspase-3 and as a diminution in the number of TUNEL- and annexin V-positive cells. This suggests that MT may be acting through the extrinsic pathway or regulating the inflammatory response [32], which in turn may reduce the death receptor-induced apoptosis [33]. This explanation remains speculative, until the effects of MT on inflammatory response after SCI might be characterized in future experiments.

On the other hand, this study demonstrated that MT reduces the activity of caspase-3 only in the early stage of apoptosis (24 h). It is known that this protease is involved in the two pathways that lead to cell death by apoptosis, the intrinsic or mitochondrial glutamate receptors activated by quinolinic (QUIN) and kainic (KA) acids [34]. The extrinsic pathway is activated by death receptors, where TNF acts as an effector of the processes of chromatin condensation and DNA fragmentation [35]. Reduction of caspase-3 activity may be the result of the ability of MT to diminish microglial activation and QUIN production, as demonstrated by Chung et al. [36] in a brain trauma model. They found that the neurotoxic kynurenine pathway intermediate QUIN is rapidly produced, within 24 h, by reactive microglia after trauma, and exogenously administered MT attenuates this effect. Recently, Leung et al. [37] showed that exogenous MT-II acts via the LRP1 receptor to alter the inflammatory response of microglia following TNF*α* stimulation, providing a more supportive environment for axon growth. Similarly, we observed that MT decreases the activity of caspase-3 in the sham group, suggesting that this protein can cross the blood-brain barrier to induce a cytoprotective effect. The exact mechanism by which it could be transported through this barrier has not yet been elucidated; however, it is thought that it could be transported through the low-density lipoprotein megalin receptor [38].

In this study, we observe that several cells were colocalizing TUNEL (green color, figure) and propidium iodide (orange color, figure), indicating the coincidence of necrotic cell death; based on the classical definition and the morphological criteria of necrosis, this is the most frequent mechanism by which nerve cells die by excitotoxicity. Under certain conditions, necrotic cells might also show fragmentation of DNA [33] and TUNEL-positive staining. In those cases, both cell death processes may be occurring simultaneously. In our study, it was not possible to rule out that positivity to the TUNEL found in the lesion area is related to both death processes [39]. The classic division between apoptosis and necrosis has been discussed, and a model that proposed a continuity is suggested, between the classical pathway of apoptosis mediated by caspases and necrosis or cell lysis [40]. The intermediate steps that pose would be the following: (a) programmed cell death similar to apoptosis, (b) cell death independent of caspases, and (c) similar programmed cell death to necrosis. Especially this criterion is important in the analysis of cell death that occurs in neurological processes as well as spinal cord injury.

In this study, we observed a reduction of positive cells to annexin V and TUNEL due to the effect of MT in both early and late stages of apoptosis. Previous research has shown the presence of cell death by apoptosis in neurons, oligodendrocytes, microglia, and astrocytes after an SCI [1, 41, 42]. Liu et al. [43], using specific markers to differentiate between neurons and glia, demonstrated the presence of TUNEL-positive cells from 4 h to 14 days in groups of both neurons and glial cells after an SCI. Likewise, they observed that the number of positive cells increases with time in the periphery of the lesion, concluding that cell death by apoptosis contributes to neuronal and glial death, after an SCI, and that the process promotes the expansion of the damage from the epicenter towards the periphery. In the same way, our results agree with those obtained by Penkowa et al. [44], in a model of brain damage induced by kainic acid (KA). In that study, the authors used a transgenic mouse overexpressing MT, and they found a decreased number of positive cells to TUNEL, three days after treatment with KA, as compared to the group without MT overexpression. In the same direction, they observed a lower neurodegeneration of CA3 cells of the hippocampus, confirming a neuroprotective effect of the MT in that model. Recently, Prado et al. [45] demonstrated an increase of TUNEL-positive cells in animals deficient to MT in a cryolesion model of the cerebral cortex.

Despite being different models of damage, those results confirm an antiapoptotic function of MT-II in various models of cell death. Likewise, when the rats were administered with MT, a better preservation of neuronal prolongations was observed, which were visible using the antibody against neurofilament [46], in according with the present work, a greater number of immunoreactive neurofilament fibers in the groups treated with MT are shown qualitatively (Figure 6). This finding is in addition to those previously observed by various groups in which they propose MTs as possible neuroprotective proteins in models of damage to the nervous system including focal brain injury [15], focal cerebral ischemia [47], and spinal cord injury [48]. Regarding neural regeneration, it has been proposed that MT promotes neuritic elongation and increases the axonal size in the cortex of damaged adult rats [46] and promotes neuritic growth in retinal ganglion cells [49]. The antiapoptotic mechanism of MT is unknown; however, it can be hypothesized that, after a mechanical or cytotoxic damage to neurons, several factors are activated that induce the expression of MT-I/-II, mainly in adjacent astrocytes. MT-I/-II is released, both actively and passively by astrocytes into the extracellular space near the lesion. Those MT deposits can interact with low-density lipoprotein receptors, LRP1 and LRP2 (megalin), and activate signal transduction involving both the MAPK and PI3k/Akt pathways and the CREB growth factor, which acts to promote neuronal survival and neuritic growth. Interestingly, dual activation of those signaling pathways is generally associated to the activation of growth factor receptors [50] and it has been observed that the expression of MT-I/-II increases rapidly (up to 8 times) after an SCI [51], and the total expression of MT proteins increases in a biphasic manner at 4 and 24 h after an SCI in rats [19], making those proteins an early line of defense against spinal cord damage. Finally, the findings of this work are important in understanding what mechanisms are acting after MT induction by lesion that can improve motor function and increase the amount of tissue preserved after an SCI, as demonstrated previously by our group [48].

## 5. Conclusion

Our findings demonstrate that exogenously administered metallothionein is an effective antioxidant and antiapoptotic treatment decreasing the extrinsic pathway (initiated by inflammation) and resulting in a diminished cell death after a traumatic spinal cord injury.

## Data Availability

Data available on request to the corresponding author.
